# Anxiety and depression in working-age cancer survivors: a register-based study

**DOI:** 10.1186/s12885-017-3347-9

**Published:** 2017-05-19

**Authors:** Laura Inhestern, Volker Beierlein, Johanna Christine Bultmann, Birgit Möller, Georg Romer, Uwe Koch, Corinna Bergelt

**Affiliations:** 1 Department of Medical Psychology, University Medical Center Hamburg-Eppendorf, Martinistr. 52, W26, 20246 Hamburg, Germany; 2 Department of Child and Adolescent Psychiatry, Psychotherapy and Psychosomatics, University Medical Center Muenster, Muenster, Germany

**Keywords:** Anxiety, Depression, Survivor, Cancer, Oncology

## Abstract

**Background:**

Anxiety and depression can be a long-term strain in cancer survivors. Little is known about the emotional situation of cancer survivors who have to deal with work- and family-related issues. The purpose of this study was to investigate anxiety and depression in working-age cancer survivors and associated factors.

**Methods:**

A register-based sample of 3370 cancer survivors (25 to 55 years at time of diagnosis) diagnosed up to six years prior to the survey was recruited from two German cancer registries. Demographic and medical characteristics as well as self-reported measures were used.

**Results:**

Overall, approximately 40% of the survivors reported moderate to high anxiety scores and approximately 20% reported moderate to high depression scores. Compared to the general population, working-age cancer survivors were more anxious but less depressed (*p* < .001). Subgroups with regard to time since diagnosis did not differ in anxiety or depression. Anxiety and depression in cancer survivors were associated with various variables. Better social support, family functioning and physical health were associated with lower anxiety and depression.

**Conclusions:**

Overall, we found higher anxiety levels in cancer survivors of working-age than in the general population. A considerable portion of cancer survivors reported moderate to high levels of anxiety and depression. The results indicate the need for psychosocial screening and psycho-oncological support e.g. in survivorship programs for working-age cancer survivors. Assessing the physical health, social support and family background might help to identify survivors at risk for higher emotional distress.

## Background

Cancer patients display higher levels of anxiety and depression compared to the general population [1]. In particular, newly diagnosed cancer patients and patients under treatment, such as chemotherapy or radiation, are emotionally distressed [2–4].

The increase of the 5-years survival rate during the last decades contributes to a higher rate of cancer patients becoming long-term survivors and dealing with side effects of treatment and diagnosis [5]. Additionally to physical consequences, some studies suggest increased levels of anxiety and depression even years after diagnosis [6, 7]. Other studies report low levels of depression and anxiety [8, 9]. So far, in most studies on anxiety and depression the mean age of participants was 55 years or older [2–4, 7, 10]. Studies with younger cancer survivors have mainly focused on breast cancer patients [6, 8, 9, 11] or testicular cancer patients [12, 13].

According to Erikson’s theory of adult development, central developmental tasks during the developmental stages from 20 to 64 years (intimacy vs. isolation, generativity vs. stagnation) are forming a close relationship, raising children or building an economic existence e.g. with regard to work [14]. A cancer disease during this period can lead to particular challenges for patients. Parenting children but also financial aspects and career changes may lead to a high pressure to get well [15, 16]. Navigating family life during the trajectory of the cancer disease demands a careful balance between the roles as patient and parent [15]. At the same time, cancer disease and treatment can affect the working ability and the reintegration into daily work after successful treatment [17].

Findings of previous studies indicate that younger cancer survivors show higher distress levels than older cancer survivors [1, 9, 11, 18]. Since cancer survivors at working-age face developmental tasks with high responsibilities such as building and caring for a family and establishing the own identity in the social and work environment [14, 19], there is a need to better understand their emotional situation. Identifying the emotional burden and characteristics of cancer survivors most at risk may allow tailored support in after care and survivorship programs to improve their situation.

The first aim of this study was to investigate the prevalence of anxiety and depression in a population-based sample of cancer survivors at working-age (25 to 55 years at the time of diagnosis) and to identify the rate of survivors with clinically relevant psychological burden during cancer survivorship. Secondly, we wanted to compare anxiety and depression in younger survivors to age- and gender-adapted normative values. Finally, we investigated socio-demographic, disease-related and family-related factors associated with anxiety and depression in cancer survivors.

## Methods

### Study design and participants

In cooperation with two regional cancer registries in northern Germany (Hamburg, Schleswig-Holstein), cancer survivors between 25 and 55 years at the time of diagnosis who were diagnosed less than six years prior to the survey were identified as potentially eligible. An information letter, a set of self-report questionnaires, a consent form and a stamped return envelope were sent to all patients. After 4 weeks, non-responders received a reminder letter.

Due to ethical considerations, patients diagnosed with cancer entities with high mortality rates (digestive organs; lower respiratory organs; eye/brain/central nervous system; secondary/ill-defined and other malignant skin neoplasms) were excluded.

Datasets provided by the cancer registries contained date of birth, cancer diagnosis and date of cancer diagnosis for all patients. UICC–staging (TNM classification) was provided for 70.2% of the patients.

The local research ethics committees approved the survey.

### Measures

We assessed socio-economic status (SES) using the Winkler-Stolzenberg index [20] including self-reported information on education and occupational qualification, job-related position and family income. The index was categorized into three levels (low, middle and high).

To assess anxiety and depression, the German Version of the Hospital Anxiety and Depression Scale (HADS-D) was used [21]. Each of the 14 items was rated from 0 to 3, and item scores were generated for the two subscales. Based on the commonly used cut-off scores, patients were assigned to the categories normal (0–7), moderate (8–10) and high (11 or above) levels of anxiety and depression [22]. The instrument shows good to very good validity and satisfactory reliability for both subscales [23].

The Oslo Social Support Scale was used to assess social support [24]. Three questions were asked: ‘How easy is it for you to get practical help from neighbours if you should need it?’ (very easy, easy, possible, difficult, very difficult), ‘How many people are so close to you that you can count on them if you have serious problems?’ (none, 1–2, 3–5, 6 or more) and ‘How much concern do people show in what you are doing?’ (a lot, some, uncertain, little, no concern). The total score was calculated by summarizing the raw scores of each item. Higher scores indicate higher social support. In our sample the internal consistency was 0.71.

Physical quality of life was measured with the physical component summary of the SF-8 Health Survey [25]. The instrument has proven to be reliable and valid [26]. The scale ranges from 0 to 100. Higher scores indicate higher physical health.

The general functioning scale of the Family Assessment Device (FAD-GF) [27] was used to measure family functioning. The general functioning scale measures the overall health and pathology of the family with regard to family functioning and shows excellent psychometric properties [27, 28]. Twelve items (6 positive and 6 negative) were rated from 1 (strongly agree) to 4 (strongly disagree), and a global family functioning score was generated with higher scores indicating worse family functioning.

### Statistical analyses

Statistical analyses were conducted using Predictive Analytics Software PASW 18.0. To investigate the prevalence of anxiety and depression in cancer survivors, we used descriptive analyses. To identify the rate of cancer survivors with clinical relevant anxiety and depression during survivorship, we used cut-off scores of the HADS [22, 29]. We conducted group comparisons between cancer survivors with regard to time since diagnosis (<2 years, 3–4 years, 5–6 years >6 years since diagnosis) using chi^2^-tests for categorical data.

We compared our sample with a representative sample of the German population (*N* = 4110, mean age 50.3 years) [29] using one-sample t-tests with age- and gender-adapted mean values. For this, we assigned an age- and gender-adapted norm value for each patient, computed the mean value and included it as the reference value.

To investigate the representativeness of the sample, responders and non-responders were compared using chi^2^-tests for categorical data and t-tests for metric data.

To identify factors associated with anxiety and depression, multiple linear regression analyses utilizing a stepwise backward method with anxiety and depression scores (HADS) as dependent variables were performed. The independent variables entered in the analyses were socio-demographic variables (age at time of the survey (in years), gender (male/female), employment status (employed/unemployed), socioeconomic status (high/middle/low), social support (OSLO)). Family-related variables entered in the analyses were living with a partner (yes/no), having a child aged ≤21 years at the time of diagnosis (yes/no) and family functioning (FAD-GF). Disease-related variables included were number of treatment modalities received (none/one/two/three), cancer diagnosis (breast/female genital organs/male genital organs/hematological/skin/other), time since diagnosis (in months) and physical health (SF-8). Nominal variables were converted into dummy variables.

To estimate effect sizes we used Cohen’s d and Cramer’s V [30]. Two-tailed significance was determined using a significance level of *p* < 0.05.

## Results

### Participants

**Fig. 1:**
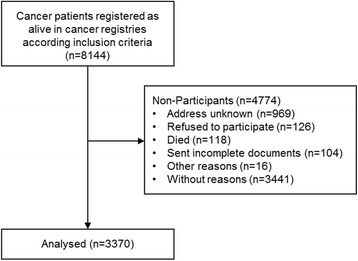
Consort flowchart of the sample

Eight thousand one hundred forty-four patients were invited to participate in the study. Four thousand seven hundred seventy-four persons did not take part in the survey for several reasons (Fig. 1). Altogether, the data from *N* = 3370 (response rate 41.3%) cancer survivors were included in the analyses (Fig. 1).

Seventy-four percent of the survivors were female; mean age was 50 years (SD 6.8). Most survivors were living with a partner (76%) and were employed in full- or part-time (72%). About half of the survivors had been diagnosed with breast cancer (52%). The mean time since diagnosis was 44 months. We found significant differences between men and women with small effect sizes for most variables. However, men were more likely to be employed in full-time (*p* < .001, V = 0.45) and belonged more often to upper socioeconomic class (*p* < .001, V = 0.16). With regard to disease-related variables men and women differed in cancer entities (*p* < .001, V = 0.79), UICC-stage (*p* < .001, V = 0.40) and kind of treatment received (*p* < .001, V = 0.18–0.33) (Table 1).

**Table 1:** Demographic and medical characteristics of a population-based sample of cancer survivors (*n* = 3370)

	total^a^		women		men		*p* ^b^	d/V ^c^
	*n*	%	*n*	%	*n*	%		
Total	3370	100	2502	74.2	868	25.8		
Mean age (SD)	50.1 (6.8)		50.0 (6.5)		50.6 (7.5)		**.016**	.085
Age, y								
≤ 30	27	0.8	15	0.6	12	1.4	**<.001**	.109
31–40	276	8.2	194	7.8	82	9.4		
41–50	1248	37.0	1001	40.0	247	28.5		
51–60	1772	52.6	1258	50.3	514	59.2		
> 60	47	1.4	34	1.4	13	1.3		
Marital status								
unmarried	474	14.1	324	13.0	150	17.3	**<.001**	.095
married	2291	68.3	1686	67.8	605	69.9		
separated/divorced	492	14.6	390	15.7	102	11.7		
widowed	96	2.9	87	3.5	9	1.0		
Living with a partner^d^	2556	76.5	1874	75.5	681	79.4	.050	-
Socioeconomic class								
low	665	20.1	497	20.2	168	19.6	**<.001**	.162
middle	1822	54.9	1447	58.8	375	43.9		
upper	829	25.0	517	21.0	312	36.5		
Employment								
Full time	1290	39.0	675	27.5	615	71.8	**<.001**	.448
Part time	1106	33.4	1053	42.9	53	6.2		
Housewife	290	8.8	278	11.3	12	1.4		
Not employed (unemployed, retired, other)	624	18.9	447	18.2	177	20.7		
Mean time since diagnosis (SD), mo	44.4 (23.3)		46.5 (27.6)		49.7 (37.2)		**.018**	.098
Diagnosis								
Breast	1757	52.1	1750	69.9	7	0.8	**<.001**	.790
Female genital organs	277	8.2	276	11.0	-	-		
Prostate	329	9.8	-	-	329	9.8		
Urinary tract	92	2.7	25	1.0	67	7.7		
Digestive organs	215	6.4	101	4.0	114	13.1		
ENT	126	3.7	37	1.5	89	10.3		
Skin	224	6.6	138	5.5	86	9.9		
Soft tissue	27	0.9	10	0.4	17	2.0		
Hematological	241	7.2	108	4.3	133	15.3		
Others	82	2.4	57	2.3	25	2.9		
UICC -Stage								
I	1063	31.5	934	37.3	129	14.9	**<.001**	.402
II	759	22.5	664	26.5	95	10.9		
III	403	12.0	330	13.2	73	8.4		
IV	141	4.2	75	3.0	66	7.6		
No information/Not determinable	1004	29.8	499	20.0	505	58.2		
Treatment								
Surgery	3132	93.6	2392	96.3	740	85.8	**<.001**	.187
Chemotherapy	1904	56.9	1559	62.7	345	40.1	**<.001**	.199
Radiotherapy	2107	63.0	1795	72.2	312	36.3	**<.001**	.325

*SD* standard deviation, *y* years, *mo* months, *ENT* ear, nose, throat, *UICC* Union for International Cancer Control

^a^Range of missing values 0 to 60

^b^ *p* value determined using chi-square test and analysis of variance, bold print indicates significance

^c^Effect sizes: d, Cohen’s d; V, Cramer’s V

^d^Living with a partner in one household irrespective of marital status

According to the large sample size, we found statistically significant differences between responders and non-responders in age (mean: 50.1 years vs 49.1 years), gender (74% women vs 66% women), diagnosis (breast cancer 52%vs 38%) and time since diagnosis (mean: 44.4 months vs 46.6 months). However, the effect sizes were small (range *d* = 0.11 to *d* = 0.15) [31].

### Prevalence of anxiety and depression

**Fig. 2:**
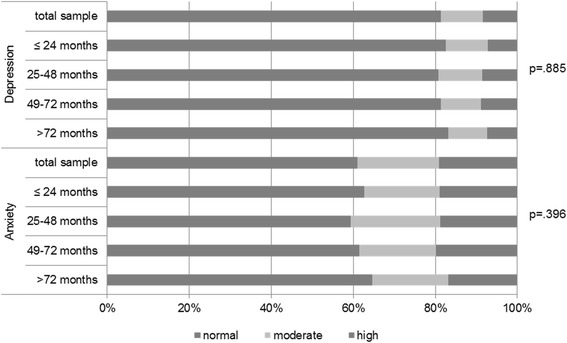
Anxiety and depression ^a^, months post-diagnosis, *n* = 3370. ^a^ According to the Cut-Off Scores of the Hospital Anxiety and Depression Scale (HADS) [22]

The mean anxiety score of the sample was 6.8 (SD = 4.1) and the mean depression score was 4.1 (SD = 4.0). In the total sample 39% of the survivors reported moderate to high anxiety scores indicating borderline or clinically relevant levels of anxiety. Nineteen percent reported moderate to high depression scores indicating borderline or clinically relevant levels of depression. In the subgroups with regard to time since diagnosis, the rate of survivors with borderline or clinically relevant anxiety scores ranged from 36 to 41%. The rate for borderline and clinically relevant anxiety scores ranged from 17 to 19%. We found no differences in any of the subscales (anxiety, depression) between survivors ≤2 years post diagnosis, survivors 3–4 years post diagnosis, survivors 5–6 years post diagnosis and survivors more than 6 years post diagnosis (Fig. 2).

In the total sample as well as in all subgroups with regard to time since diagnosis, cancer survivors reported statistically significant higher levels of anxiety and lower levels of depression compared with population-based norms (*p* = .001) (Table 2).

**Table 2:** Anxiety and depression in a population-based sample of cancer survivors compared to population-based values^a^ (*n* = 3370)

	Anxiety^b^				Depression^b^			
	Cancer survivors	Norm population^a^			Cancer survivors	Norm population^a^		
Time post diagnosis	Mean (SD)	Mean	*p* ^c^	d^d^	Mean (SD)	Mean	*p* ^c^	d^d^
≤ 24 months (*n* = 556)	6.7 (4.0)	5.05	**<.001**	.45	4.1 (4.0)	4.71	**<.001**	.16
25–48 months (*n* = 1307)	6.8 (4.2)	5.07	**<.001**	.45	4.2 (4.0)	4.71	**<.001**	.14
49–72 months (*n* = 1141)	6.8 (4.2)	5.08	**<.001**	.44	4.1 (4.0)	4.78	**<.001**	.17
> 72 months (*n* = 302)	6.5 (4.0)	5.07	**<.001**	.39	4.0 (3.8)	4.86	**<.001**	.22
Total (*n* = 3306)	6.8 (4.1)	5.07	**<.001**	.44	4.1 (4.0)	4.75	**<.001**	.16

Abbreviations: SD, standard deviation.

^a^Gender and age adapted norm values according to Hinz &Brähler [29]

^b^According to the Hospital Anxiety and Depression Scale (HADS)

^c^ *p* value determined according to single sample t-test with norm mean as reference, bold print indicates significance

^d^Effect size: Cohen’s d

### Factors associated with anxiety and depression

Stepwise-backward multiple linear regression analysis revealed that higher anxiety was statistically significant associated with female gender, younger age, less social support, diagnosis breast cancer compared to diagnosis skin cancer, lower physical health and poorer family functioning (Table 3). Being unemployed, receiving less social support, receiving no treatment, shorter time since diagnosis, lower physical health and poorer family functioning were statistically significant associated with higher depression (Table 3). Diagnoses of female or male genital organ cancer, diagnosis of hematological cancer or of the category “other cancer entity” were statistically significant associated with higher depression scores compared to survivors with breast cancer.

**Table 3:** Factors associated with anxiety and depression^a^ in younger cancer survivors^b^

	reference	β^e^	*p* ^f^
Anxiety^a^			
Female	Male	.082	<.001
Age	-	−.090	<.001
Social support^c^	-	−.130	<.001
Diagnosis skin cancer	breast	−.041	.027
Physical health	-	−.284	<.001
General family functioning	-	.300	<.001
Depression^a^			
Employed	unemployed	−.070	<.001
One treatment modality	None	−.064	.001
Two treatment modalities	None	−.034	.055
Diagnosis female genital organs	breast	.036	.029
Diagnosis male genital organs	breast	.051	.003
Diagnosis hematological cancer	breast	.032	.049
Diagnosis other cancer entity	breast	.058	.001
Time since diagnosis^d^	-	−.032	.041
Social support^c^	-	−.206	<.001
Physical health	-	−.354	<.001
General family functioning	-	.336	<.001

^a^According to the Hospital Anxiety and Depression Scale (HADS)

^b^missing values were excluded listwise, *n* = 2190 participants included into regression analyses; UICC staging was not included into analyses due to missing data in approximately 30% of the cases

^c^According to the OSLO Social Support Scale

^d^in months

^e^β value: standardized beta-coefficient

^f^ *p* value determined according to stepwise-backward linear regression

The model for depression (adjusted R^2^ = 45%) performed better than that for anxiety (adjusted R^2^ = 28%).

## Discussion

In this study, we examined anxiety and depression in a large epidemiological sample of cancer survivors of working-age. The findings of this study show that among working-age cancer survivors, about 40% reported elevated levels of anxiety and 20% elevated levels of depression. Anxiety and depression rates of survivors diagnosed 1–2 years or 3–4 years prior to the survey did not differ statistically significant from those of patients diagnosed 5–6 years prior to the survey. These findings are similar to another cross-sectional population-based study in breast cancer survivors (mean age 62 years), who also found no differences in anxiety and depression with regard to time since diagnosis [11]. However, to investigate the course and possible stability of anxiety and depression after diagnosis longitudinal studies on cancer survivors at working-age are necessary.

Depression scores in our sample were statistically significantly lower and anxiety scores were significantly higher than in the general population. Effects between groups for both subscales were small. Nevertheless, the results indicate that cancer diagnosis seems to be a long-term strain regarding anxiety in cancer survivors of working-age. This finding is in line with the conclusions of a meta-analysis in long-term cancer survivors by Mitchell and colleagues (2013) [32]. Cancer survivors may fear cancer recurrence and cancer progress, which may be a stressor even years after diagnosis [33]. Cancer survivors need to adapt to the uncertainty of the cancer disease, which may affect work life and family life [34] and can enhance anxiety. To fulfill their developmental tasks [14], cancer survivors of working-age struggle to stay in workforce, raise their children and get back to ‘normal’ life, which may lead to additional burden. At the same time, better appreciation of life, closer relationships and priority changes may help cancer survivors to overcome depressive symptoms [35].

Several demographic and disease-related variables were significantly associated with anxiety and depression (Table 3). Besides disease-related factors such as cancer entity or number of treatment modalities received, unemployment was statistically significant associated with higher depression scores. Reintegration in daily work and structured daily routines may improve the well-being of cancer patients after treatment [36, 37]. However, in our study the predictive value of employment was statistically significant but rather low. We found no associations between family-related factors such as having minor children or living with a partner and anxiety or depression. It seems that rather psychosocial factors are related to anxiety and depression. Higher social support, better physical functioning and better family functioning were the strongest predictors of lower anxiety and lower depression scores. Better physical health may help to encounter daily requirements successful and hence, rebuild daily life, structure and normalcy after diagnosis and treatment [38]. Furthermore, the results reveal the importance of familial and social background to overcome the burden of cancer disease, and illustrate the importance of supporting all family members to enhance family functioning.

There are some limitations in the current study. Because we used register-based recruitment, all patients were contacted without previous reference to our institution. Nevertheless, a response-rate of 41% was reached, which is similar to other register-based studies [39, 40]. Comparing responders and non-responders, in our sample women and breast cancer survivors are slightly overrepresented and responders are marginally older than non-responders, which may lead to limitations with regard to representativeness of our sample and generalizability of the findings. However, effect sizes of the differences were small. Moreover, UICC staging was only provided for approximately 70% of the patients, and therefore, it was not included in the regression analyses.

Due to ethical reasons, we did not include cancer patients with diagnoses with a high mortality rate in our survey. To prevent families from an additional burden where a patient might have recently died or is in the palliative stage, we excluded tumor diagnoses with poor 5-year-survival rates such as lung cancer and brain tumors. Previous studies assessing anxiety and depression with the HADS found that lung cancer patients and advanced cancer patients reported higher rates of depression than in our sample [2, 41–44]. Therefore, our study might underestimate anxiety and depression in working-age cancer survivors. With regard to the use of the HADS as assessment instruments, most analyses confirm the two-factorial structure, while some studies suggest other factorial solutions [29, 45]. Moreover, the thresholds for clinical elevated levels of anxiety and depression vary between studies [46]. Since there are no normative data and final solutions, we decided to use the established subscales anxiety and depression with the commonly used cut-off scores [22, 29].

Finally, as we conducted the study in Germany, the results can only be generalized to countries with socialized medicine. Cancer survivors in countries without socialized medicine may experience other stressors and challenges with regard to child rearing and work.

## Conclusions

Dealing with cancer during working-age can be challenging and enhance levels of anxiety and depression. Our findings show that even up to six years after diagnosis survivors report elevated levels of anxiety compared to norm values. The results indicate the necessity of psychological screening up to years after diagnosis to identify cancer survivors at risk and may give implications for survivorship programs adjusted to the situation of cancer survivors at working-age. Additionally to physical health, survivorship programs should address psychosocial issues such as social support and family background.

## Abbreviations

FAD-GF: Family Assessment Device – General Functioning Scale
HADS-D: Hospital Anxiety and Depression Scale – German Version
PASW: Predictive Analytics Software
SES: Socio-economic status
TNM: Tumor, nodes, metastases classification
UICC: Union for International Cancer Control
